# The Impact of Prostate-Specific Antigen Screening on Prostate Cancer Incidence and Mortality in China: 13-Year Prospective Population-Based Cohort Study

**DOI:** 10.2196/47161

**Published:** 2024-01-18

**Authors:** Xiaohao Ruan, Ning Zhang, Dawei Wang, Jingyi Huang, Jinlun Huang, Da Huang, Tsun Tsun Stacia Chun, Brian Sze Ho Ho, Ada Tsui-Lin Ng, James Hok-Leung Tsu, Yongle Zhan, Rong Na

**Affiliations:** 1 Department of Urology Ruijin Hospital Shanghai Jiao Tong University School of Medicine Shanghai China; 2 Department of Surgery LKS Faculty of Medicine The University of Hong Kong Hong Kong China (Hong Kong); 3 Division of Urology Department of Surgery Queen Mary Hospital Hong Kong China (Hong Kong)

**Keywords:** prostate-specific antigen, PSA, prostate cancer, prostate screening, screening interval, incidence, mortality, cohort study, electronic health record, China

## Abstract

**Background:**

The status of prostate-specific antigen (PSA) screening is unclear in China. Evidence regarding the optimal frequency and interval of serial screening for prostate cancer (PCa) is disputable.

**Objective:**

This study aimed to depict the status of PSA screening and to explore the optimal screening frequency for PCa in China.

**Methods:**

A 13-year prospective cohort study was conducted using the Chinese Electronic Health Records Research in Yinzhou study’s data set. A total of 420,941 male participants aged ≥45 years were included between January 2009 and June 2022. Diagnosis of PCa, cancer-specific death, and all-cause death were obtained from the electronic health records and vital statistic system. Hazard ratios (HRs) with 95% CIs were estimated using Cox regression analysis.

**Results:**

The cumulative rate of ever PSA testing was 17.9% with an average annual percent change (AAPC) of 8.7% (95% CI 3.6%-14.0%) in the past decade in China. People with an older age, a higher BMI, higher waist circumference, tobacco smoking and alcohol drinking behaviors, higher level of physical activity, medication use, and comorbidities were more likely to receive PSA screening, whereas those with a lower education level and a widowed status were less likely to receive the test. People receiving serial screening ≥3 times were at a 67% higher risk of PCa detection (HR 1.67; 95% CI 1.48-1.88) but a 64% lower risk of PCa-specific mortality (HR 0.36; 95% CI 0.18-0.70) and a 28% lower risk of overall mortality (HR 0.72; 95% CI 0.67-0.77). People following a serial screening strategy at least once every 4 years were at a 25% higher risk of PCa detection (HR 1.25; 95% CI 1.13-1.36) but 70% (HR 0.30; 95% CI 0.16-0.57) and 23% (HR 0.77; 95% CI 0.73-0.82) lower risks of PCa-specific and all-cause mortality, respectively.

**Conclusions:**

This study reveals a low coverage of PSA screening in China and provides the first evidence of its benefits in the general Chinese population. The findings of this study indicate that receiving serial screening at least once every 4 years is beneficial for overall and PCa-specific survival. Further studies based on a nationwide population and with long-term follow-up are warranted to identify the optimal screening interval in China.

## Introduction

Globally, prostate cancer (PCa) is the second-most commonly diagnosed male cancer, with an estimated 1.41 million new cases and 0.37 million deaths reported in 2020 [1]. In China, among all cancers, PCa ranks sixth in incidence and seventh in mortality [2], which have been increasing rapidly over the past decades [3]. The aging population and the widespread use of prostate-specific antigen (PSA) testing could partially explain the significant increase in disease burden.

The uptake of PSA testing has been well characterized in Western countries. In Switzerland, for example, the coverage rate of PSA screening has increased to 70% [4]. In the United Kingdom, 55.3% of men aged 40-75 years have undergone at least 1 PSA screening [5]. In the United States, 32.1% of men aged 50 years or older have had PSA screening for routine reasons [6]. On the contrary, anecdotal evidence suggests a low PSA screening rate in most Asian countries [7]. A wide range of factors have been associated with screening behavior in the general population. For instance, people with an older age, a family history of PCa, and a high BMI were more likely to receive PSA testing, whereas socioeconomic deprivation, heavy smoking, and comorbid diabetes were associated with a lower likelihood of PSA testing [5].

PSA screening policy and guidelines to date have been well established in the West, including the recommendation statement of screening for prostate cancer by the US Preventive Services Task Force [8], the National Comprehensive Cancer Network’s guidelines for Prostate Cancer Early Detection [9], European Association of Urology–European Association of Nuclear Medicine–European Society for Radiotherapy & Oncology–European Society of Urogenital Radiology–International Society of Geriatric Oncology Guidelines on Prostate Cancer [10]. However, PSA screening also leads to overdiagnosis and overtreatment of insignificant PCa, which has been increasingly criticized [11]. Several large randomized controlled trials (RCTs) have suggested that such screening contributes to little benefit for overall and cancer-specific survival, especially when taking its efficiency or cost-effectiveness into account [12]. In China, the effect of PSA screening is poorly understood, and few epidemiological studies have depicted the rate and trend of PSA testing from a population-based perspective.

On the other hand, a consensus on screening strategy has yet to be determined. Some studies suggest that one-time PSA screening is not beneficial [13], while others suggest that annual screening is not cost-effective [14]. The optimal frequency and interval for serial screening remains to be concurred. Kobayashi et al [15] reported an appropriate interval of ≥3 years for PSA rescreening, while Shao et al [16] concluded that more frequent PSA testing could aggravate the risk of overdiagnosis. In well-known clinical trials, no consensus on screening intensity has been established. For example, one-time screening was applied in the Cluster Randomised Trial of PSA Testing for Prostate Cancer; annual screening was undertaken in the Prostate, Lung, Colorectal and Ovarian Cancer Screening trial; and screening once every 2-4 years was performed in the European Randomised Study of Screening for Prostate Cancer [12].

Given a paucity of evidence regarding the depiction of PSA testing, and the benefits of different screening strategies in China, we performed a population-based prospective cohort study using electronic health records (EHRs) data. The primary aims of this study are to evaluate the effects of PSA screening and to explore the optimal screening frequency and interval in the Chinese population.

## Methods

### Study Setting and Population

Data for this study were obtained from the Chinese Electronic Health Records Research in Yinzhou (CHERRY) study, the first population-based cohort study linking big data of integrated individual-level EHRs. Detailed information regarding the study design, inclusion criteria, data collection, and procedure of the study is published previously [17]. Briefly, the CHERRY study to date has comprised a total of more than 1 million permanent residents living in Yinzhou, an economically advanced region of southeastern China. Participants were included in the CHERRY study if they (1) registered in the system, (2) were older than 18 years on January 1, 2009, (3) were living in Yinzhou for more than 6 months, and (4) provided consent to participate in the study. The data set of the CHERRY study was integrated from the population census, primary care, electronic medical records, health check, surveillance, and vital statistic, where all health-related activities (eg, inpatient and outpatient visits) within the region were recorded.

**Figure 1:**
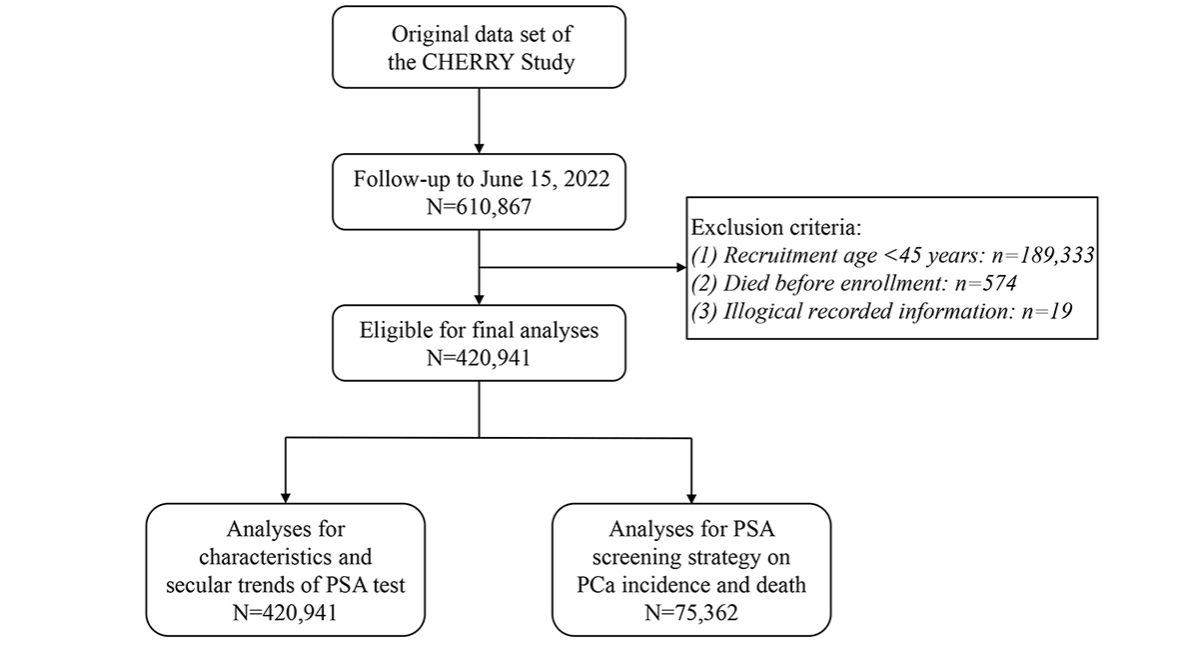
Flowchart for participant recruitment in this study. CHERRY: Chinese Electronic Health Records Research in Yinzhou; PCa: prostate cancer; PSA: prostate-specific antigen.

As 45 years is the initiation age for PCa screening recommended by the National Cancer Center of China [18], we accordingly included male participants aged ≥45 years in this study. We further excluded those (1) aged <45 years at recruitment (n=189,333), (2) who consented to participate but died before enrollment in the cohort (n=574), and (3) with illogical recorded information (eg, recorded death occurred before the uptake of PSA testing; n=19); 420,941 eligible participants were retained in the final analysis. A 3×3 matrix data quality assessment framework proposed by Weiskopf et al [19] was further applied to comprehensively evaluate the data quality of the data set. A detailed data quality assessment report is presented in Multimedia Appendix 1. The flowchart of this study is shown in Figure 1.

### PSA Screening

Serum PSA was tested using an immunoenzymatic assay with the World Health Organization’s international standard or the Hybritech Standard based on different protocols among hospitals [20]. The value and date of each PSA test were extracted from the CHERRY study’s data set. We then developed a series of variables related to PSA screening. Ever screening was defined as ever having any PSA test or not (ever vs never). The subsequent variables were related to screening strategy and were thus restricted to those ever-having a PSA test. Number of PSA screenings was defined as a total count of screenings during the follow-up years; this variable was further categorized into 3 groups (1, 2, and ≥3) for comparison. Four variables were proxies of screening frequency: receiving 1 PSA test at least annually (yes or no), receiving 1 PSA test at least biennially (yes or no), receiving 1 PSA test at least triennially (yes or no), and receiving 1 PSA test at least quadrennially (yes or no). Screening interval was defined as the average interval of having serial tests (irregular, 1 year, 2 years, 3 years, and 4 years). Detailed definitions of the abovementioned variables are provided in Table S1 in Multimedia Appendix 1.

### Outcomes of Interest

Participants were followed up for morbidity and mortality using records linked with the regional system of disease surveillance, chronic disease management, and EHRs based on diagnostic codes from the *ICD-10* (*International Statistical Classification of Diseases, Tenth Revision*). The primary outcomes of this study were prostate cancer (C61) and PCa-specific death. The secondary outcome was all-cause death.

### Covariates

Sociodemographics (age, education level, and marital status), anthropometric factors (BMI and waist circumference [WC]), lifestyle (smoking, drinking, and physical activity [PA]), use of medications (5-α reductase inhibitors), and comorbidity were included and used for adjustment in the analyses. Detailed definitions of the covariates are provided in Table S1 in Multimedia Appendix 1.

### Statistical Analysis

Descriptive statistics were summarized using means (SD) or median (IQR) values for continuous variables (normally or nonnormally distributed) and number (percentage) values for categorical variables. Follow-up person-years (PYs) were calculated from the date of cohort inception for the nonscreening group and the date of the initial PSA test for the screening group to either the date of PCa diagnosis, death, loss to follow-up, or June 15, 2022, whichever occurred first.

Temporal trends in the annual rate of first PSA screening were estimated by joinpoint regression models [21]. The direction and magnitude of the trends were assessed by the annual percentage change (APC) and average annual percent change (AAPC) with corresponding 95% CIs. Missing values were presumed to be missing at random and were filled by multiple imputations based on chained equations. Cox proportional hazards models were used to estimate the hazard ratios (HRs) and 95% CIs for PSA screening on PCa incidence, cause-specific mortality, and overall mortality. The proportional hazards assumption was tested on the basis of Schoenfeld residuals. Cox models were stratified by age-at-risk of PCa or death accordingly (5-year intervals; model 1), and HRs were adjusted for sociodemographics, anthropometric factors, lifestyle, medications, and comorbidities (model 2), and were additionally adjusted for baseline PSA values and age at the first PSA test (model 3) according to the proposed directed acyclic graph (Figure S1 in Multimedia Appendix 1). In addition, the E-value was calculated to assess the robustness of the main results against unmeasured confounders [22].

All *P* values were 2-sided and were considered significant when less than .05. The abovementioned analyses were performed using Stata (version 17.0; StataCorp), Joinpoint software (version 4.8.0.1; National Cancer Institute), and R statistical software (version 4.1.2; Foundation for Statistical Computing).

### Ethical Considerations

The study was approved by the institutional review board of the University of Hong Kong (UW 22-766). All procedures were performed in accordance with the tenets of the 1964 Declaration of Helsinki and its later amendments or comparable ethical standards. Informed consent has been obtained from all participants before the study.

## Results

### Baseline Characteristics

Among the 420,941 participants included in the study, the overall mean age was 50.4 (SD 11.9) years. After a total of 3,177,289 person-years of follow-up, 2160 men were diagnosed with PCa (incidence rate 0.68 per 1000 PYs), 92 men died from PCa (cause-specific mortality rate 0.03 per 1000 PYs), and 20,781 men died from all causes (overall mortality rate 6.78 per 1000 PYs). The mean BMI was 22.9 (SD 3.1) kg/m^2^, and 1.8% (n=3900) of them had a high WC. In terms of socioeconomic factors, 3.5% (n=14,297) of men had a bachelor’s degree or above, and 92.0% (n=221,845) of them were married. Long-term medication use (n=5319, 1.9%) and comorbidities (n=33,220, 12.1%) were observed in a small proportion of men. Smoking and drinking alcohol was prevalent among 21.3% (n=48,731) and 23.1% (n=52,916), respectively. Detailed characteristics of the study participants are shown in Table 1.

**Table 1:** Characteristics of the study participants (N=420,941).

Characteristics			Values
Age (years), mean (SD)			50.4 (11.9)
**Education level, n (%)**			
	Bachelors and above	14,297 (3.5)	
	Below bachelors	394,644 (96.5)	
	Missing	12,000 (N/A^a^)	
**Marital status, n (%)**			
	Single	3118 (1.3)	
	Married	221,845 (92.0)	
	Widowed	14,440 (6.0)	
	Divorced	1756 (0.7)	
	Missing	179,782 (N/A)	
**Medication use, n (%)**			
	No	270,225 (98.1)	
	Yes	5319 (1.9)	
	Missing	145,397 (N/A)	
**Comorbidity, n (%)**			
	No	242,324 (87.9)	
	Yes	33,220 (12.1)	
	Missing	145,397 (N/A)	
BMI (kg/m^2^), mean (SD)			22.9 (3.1)^b^
**High waist circumference, n (%)**			
	No	216,335 (98.2)	
	Yes	3900 (1.8)	
	Missing	200,706 (N/A)	
**Physical activity, n (%)**			
	Never	9056 (7.0)	
	Occasional (<1 per week)	39,388 (30.3)	
	Frequent (≥1 per week)	81,561 (62.7)	
	Missing	290,936 (N/A)	
**Tobacco smoking, n (%)**			
	Never	180,585 (78.7)	
	Ever	48,731 (21.3)	
	Missing	191,625 (N/A)	
**Alcohol drinking, n (%)**			
	Never	176,047 (76.9)	
	Ever	52,916 (23.1)	
	Missing	191,978 (N/A)	

^a^N/A: not applicable.

^b^BMI values missing for 210,431 individuals.

### PSA Screening

Overall, 17.9% (n=75,362) of participants ever attended PSA testing. Among them, 51.1% (n=38,527), 22.1% (n=16,686), and 26.7% (n=20,149) underwent testing 1, 2, and ≥3 times; two-thirds of them (63.2%, n=47,654) attended PSA screening without regularity; about one-sixth of them (16.8%, n=12,686) had a baseline PSA value of 3 ng/mL or above and had their first test at the age of 75 years or older (17.9%, n=13,514). A detailed description of PSA screening by age stratification is provided in Table 2.

During 2010-2021, the annual rates of first PSA testing ranged from 0.85% to 3.45%. Joinpoint regression analyses suggested a significant increasing tendency of uptake of PSA testing during the past 12 years (AAPC 8.70%; 95% CI 3.61-14.04; *P*=.001). The increase in trend in the later 6 years (APC 15.65%; 95% CI 7.97-23.88; *P*=.002) was 5-fold faster than that in the prior 6 years (APC 3.23%; 95% CI –5.58 to 12.86; *P*=.43; Figure S2 in Multimedia Appendix 1).

**Figure 2:**
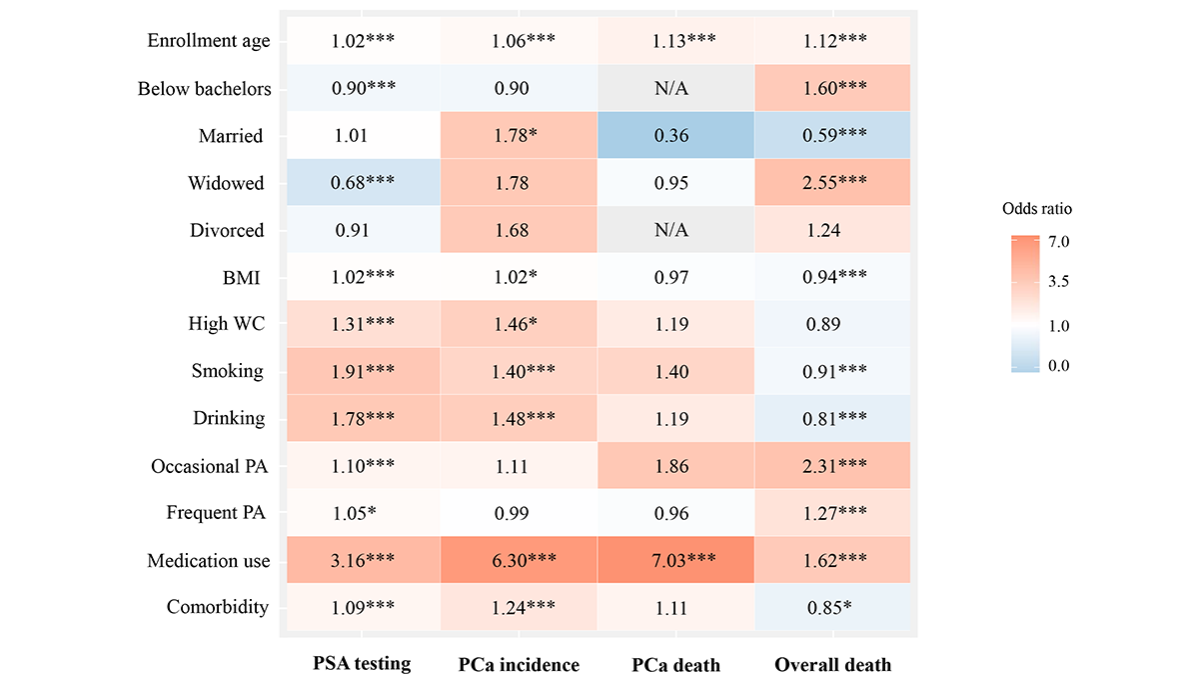
Factors related to prostate-specific antigen screening and prostate cancer incidence and mortality. PA: physical activity; PCa: prostate cancer; PSA: prostate-specific antigen; WC: waist circumference; *: *P*<.05; **: *P*<.01; ***: *P*<.001.

Figure 2 shows several common factors that could influence PSA screening behavior. Age (HR 1.02; 95% CI 1.02-1.02; *P*<.001), BMI (HR 1.02; 95% CI 1.01-1.02; *P*<.001), high WC (HR 1.31; 95% CI 1.23-1.40; *P*<.001), tobacco smoking (HR 1.91; 95% CI 1.88-1.95; *P*<.001), alcohol drinking (HR 1.78; 95% CI 1.74-1.81; *P*<.001), physical activity (HR 1.10, 95% CI 1.06-1.15; *P*<.001 for occasional PA and HR 1.05; 95% CI 1.01-1.09; *P*=.01 for frequent PA), medication use (HR 3.16, 95% CI 3.02-3.30; *P*<.001), and comorbidity (HR 1.09; 95% CI 1.07-1.10; *P*<.001) were associated with higher probability of receiving a PSA test, while those with lower education levels (HR 0.90; 95% CI 0.85-0.95; *P*<.001) and with a widowed status (HR 0.68; 95% CI 0.61-0.76; *P*<.001) may be less likely to receive a PSA test.

**Table 2:** Age-specific characteristics of participants (N=420,941) having undergone prostate-specific antigen (PSA) screening.

Characteristics		Total, n (%)	Enrollment age (years), n (%)						
			45-49	50-54	55-59	60-64	65-69	70-74	≥75
Sample size		420,941 (100)	104,178 (24.7)	88,357 (21.0)	72,689 (17.3)	56,275 (13.4)	36,476 (8.7)	24,898 (5.9)	38,068 (9.0)
**PSA screening**									
	Never	345,579 (82.1)	90,576 (86.9)	74,520 (84.3)	59,402 (81.7)	44,296 (78.7)	27,952 (76.6)	18,681 (75.0)	30,152 (79.2)
	Ever	75,362 (17.9)	13,602 (13.1)	13,837 (15.7)	13,287 (18.3)	11,979 (21.3)	8524 (23.4)	6217 (25.0)	7916 (20.8)
**Number of PSA screenings^a^**									
	1	38,527 (51.2)	7465 (54.9)	7541 (54.5)	6973 (52.5)	5947 (49.6)	4093 (48.0)	2861 (46.0)	3647 (46.1)
	2	16,686 (22.1)	2950 (21.7)	3054 (22.1)	2892 (21.8)	2652 (22.1)	1917 (22.5)	1397 (22.5)	1824 (23.0)
	≥3	20,149 (26.7)	3187 (23.4)	3242 (23.4)	3422 (25.8)	3380 (28.2)	2514 (29.5)	1959 (31.5)	2445 (30.9)
**Screening interval**									
	Irregular^b^	47,654 (63.2)	8658 (63.7)	8896 (64.3)	8460 (63.7)	7474 (62.4)	5346 (62.7)	3846 (61.9)	4974 (62.8)
	Quadrennial	3650 (4.8)	555 (4.1)	565 (4.1)	656 (4.9)	605 (5.1)	433 (5.1)	355 (5.7)	481 (6.1)
	Triennial	6967 (9.2)	1093 (8.0)	1222 (8.8)	1155 (8.7)	1201 (10.0)	788 (9.2)	646 (10.4)	862 (10.9)
	Biennial	12,409 (16.5)	2630 (19.3)	2295 (16.6)	2096 (15.8)	1876 (15.7)	1351 (15.8)	962 (15.5)	1199 (15.1)
	Annual	4682 (6.2)	666 (4.9)	859 (6.2)	920 (6.9)	823 (6.9)	606 (7.1)	408 (6.6)	400 (5.1)
**Baseline PSA value^a^ (ng/mL)**									
	<3	62,676 (83.2)	12,792 (94.0)	12,562 (90.8)	11,430 (86.0)	9853 (82.3)	6459 (75.8)	4491 (72.2)	5089 (64.3)
	≥3	12,686 (16.8)	810 (6.0)	1275 (9.2)	1857 (14.0)	2126 (17.7)	2065 (24.2)	1726 (27.8)	2827 (35.7)
**Age at first PSA test^a^, year**									
	<75	61,848 (82.1)	13,602 (100.0)	13,837 (100.0)	13,287 (100.0)	11,175 (93.3)	6281 (73.7)	2976 (47.9)	690 (8.7)
	≥75	13,514 (17.9)	0 (0)	0 (0)	0 (0)	804 (6.7)	2243 (26.3)	3241 (52.1)	7226 (91.3)

^a^Statistics were restricted to those who ever had a PSA screening.

^b^Irregular screening referred to attending screening without regularity over time.

### Effects of PSA Screening in the General Population

Compared to nonattendees, screening attendees were at 70-, 16-, and 6-fold higher risk of PCa incidence, PCa death, and overall death, respectively, in the crude model (Tables S2-S4 in Multimedia Appendix 1). After adjustment for age, education, marital status, BMI, high WC, smoking, drinking, PA, medication use, and comorbidity, we found that attendees were still at 35-, 11-, and 1.2-fold higher risk of PCa incidence (HR 35.87; 95% CI 31.82-40.85; *P*<.001), PCa death (HR 11.94; 95% CI 7.69-18.54; *P*<.001), and overall death (HR 1.27; 95% CI 1.20-1.35; *P*<.001), respectively.

### PSA Screening Strategy and PCa Incidence

**Figure 3:**
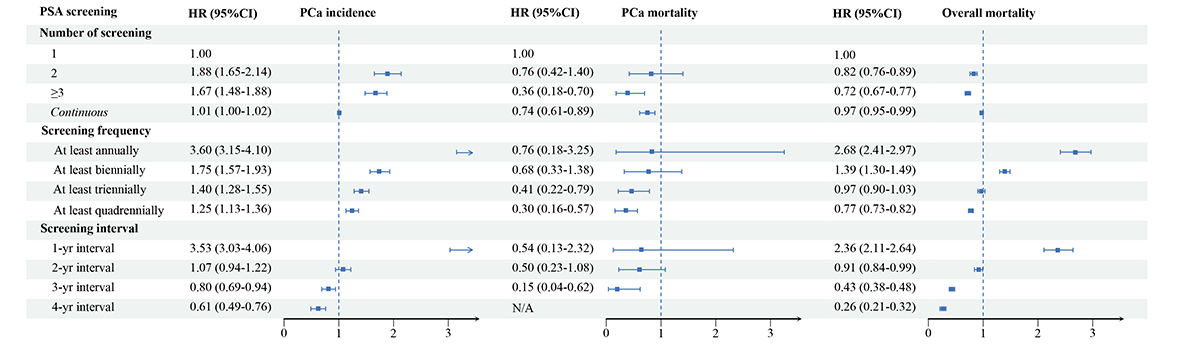
Adjusted Cox regression model for prostate-specific antigen screening strategy on prostate cancer (PCa) incidence, PCa-specific mortality, and overall mortality. HR: hazard ratio; PSA: prostate-specific antigen.

All statistics on screening strategy were restricted to individuals ever having had a PSA test (n=75,362). In the full adjustment model, people receiving a PSA test more than 3 times were at a 67% higher risk of PCa detection than those having had a PSA test once (HR 1.67; 95% CI 1.48-1.88; *P*<.001). Frequent attendees were at a higher risk of PCa detection (HR 3.60; 95% CI 3.15-4.10; *P*<.001 for having a PSA test at least annually; HR 1.75; 95% CI 1.57-1.93; *P*<.001 for having a PSA test at least biennially; HR 1.40; 95% CI 1.28-1.55; *P*<.001 for having a PSA test at least triennially; and HR 1.25; 95% CI 1.13-1.36; *P*<.001 for having a PSA test at least quadrennially). People receiving serial screening with a 1-year interval were at a higher risk of PCa detection (HR 3.53; 95% CI 3.03-4.06; *P*<.001), but those with a 2-year interval were at no significant risk of PCa detection (HR 1.07; 95% CI 0.94-1.22; *P*=.29), while those with 3- and 4-year intervals were at a lower risk of PCa detection (HR 0.80; 95% CI 0.69-0.94; *P*=.006 for a 3-year interval; HR 0.61; 95% CI 0.49-0.76; *P*<.001 for a 4-year interval; Figure 3; Table S5 in Multimedia Appendix 1).

### PSA Screening Strategy and PCa-Specific Mortality

People receiving PSA screening more than 3 times were at a lower risk of PCa-specific mortality than those receiving PSA testing once (HR 0.36; 95% CI 0.18-0.70; *P*=.002). We further found a 26% decreased risk with every additional PSA screenings (HR 0.74; 95% CI 0.61-0.89; *P*=.001). Frequent attenders were at a 24%-70% lower risk of PCa death (HR 0.76; 95% CI 0.18-3.25; *P*=.72 for at least an annual PSA screening; HR 0.68; 95% CI 0.33-1.38; *P*=.29 for at least a biennial screening; HR 0.41; 95% CI 0.22-0.79; *P*=.007 for at least a triennial screening; HR 0.30; 95% CI 0.16-0.57; *P*<.001 for at least a quadrennial screening). People receiving serial screening with a 3-year interval were at a significant lower risk of PCa death than those with other intervals (HR 0.15; 95% CI 0.04-0.62; *P*=.009). Such significance was not identified in other interval groups due to low statistical power (Figure 3 and Table S6 in Multimedia Appendix 1).

### PSA Screening Strategy and Overall Mortality

People receiving PSA screening more than 3 times were at a lower risk of overall death than those receiving PSA screening once (HR 0.72; 95% CI 0.67-0.77; *P*<.001). Furthermore, we found a 3% lower risk with every additional PSA screenings (HR 0.97; 95% CI 0.95-0.99; *P*=.001). People receiving at least annual (HR 2.68; 95% CI 2.41-2.97; *P*<.001) and biennial PSA screening (HR 1.39; 95% CI 1.30-1.49; *P*<.001) were at a higher risk of overall death, while those receiving at least triennial (HR 0.97; 95% CI 0.90-1.03; *P*=.28) and quadrennial PSA screening (HR 0.77; 95% CI 0.73-0.82; *P*<.001) were at lower risks of overall death. Compared to people with irregular screening intervals, those with a 1-year interval were at a 136% higher risk of overall death (HR 2.36; 95% CI 2.11-2.64; *P*<.001), while those with extended intervals were at a 9%-74% lower risk of overall death (HR 0.91; 95% CI 0.84-0.99; *P*=.03 for those with a 2-year interval; HR 0.43; 95% CI 0.38-0.48; *P*<.001 for those with a 3-year interval; HR 0.26; 95% CI 0.21-0.32; *P*<.001 for those with a 4-year interval; Figure 3 and Table S7 in Multimedia Appendix 1).

## Discussion

This prospective cohort study including 420,941 male participants suggests that the cumulative rate of ever PSA testing was 17.9% in the past decade, with an upward secular trend in China. Evidence provided by this study supports the benefit of regular screening (in particular, at least once every 4 years) for overall and cancer-specific survival among individuals attending repeated PSA screening.

This study revealed a low coverage of PSA screening in the Chinese population, which was consistent with the results of several prior studies. So et al [23] reported a 9.5% uptake rate of PSA testing among men in Hong Kong, while Lin et al [24] reported a proportion of 29.4% of men in Taiwan who had self-reported having received a PSA test. Lacking knowledge of or misconceptions about PCa and PSA screening, as well as insufficient health promotion, may account for the issue of low PSA uptake coverage in China [23]. As for other Asian countries, except for Japan [25], the uptake rate of PSA screening was at a similarly low level (eg, 16.9% in Iran and 0.4% in the Philippines) [26,27]. Some studies indicate that PSA testing behavior is highly determined by a variety of demographics and lifestyle factors [23,28]. In this study, we identified that Chinese men with older age, a higher BMI, high WC, smoking behavior, drinking behavior, occasional PA, medication use, and comorbidity were more likely to undergo PSA testing, whereas those with lower education background and under widowed were less likely to have undergone PSA testing. These findings help to depict a clearer picture of the status and correlates of PSA testing behavior, which provides important credence for PSA screening practice in China.

We did not find a survival benefit of PSA screening uptake in the general population as well as among annual screeners in this cohort study. One explanation was the self-selection bias [29]. People who attended PSA screening, particularly annual attendees, were more likely to be older, have unhealthy lifestyles, and be in poor physical condition; in turn, these factors were associated with increased PCa mortality. Furthermore, some nonattenders may be potential later screening attendees should they live longer or have long-term follow-up. The existence of immortal time bias influenced the calculation of true effects on PSA screening [30].

Evidence regarding the optimal screening frequency or interval remains unclear worldwide. In general, annual screening was recommended by guidelines in the United States, while a 2-year interval in Australia, a 3-year interval in Japan, and a 4-year interval in Canada [31]. In Europe, even an 8-year interval was proposed for those who were not at risk [10]. In China, the Anti-Cancer Association Genitourinary Cancer Committee recommends a 2-year interval for serial PSA screening [32]. Several studies have supported the benefit of a moderate expansion for the screening interval. Gulati et al [33] reported that a biennial screening could reduce 2.4% of overdiagnoses and half of the false-positive rate. Leeuwen [34] stated that a 2-year screening interval significantly decreased the advanced PCa incidence. Heijnsdijk [14] indicated that screening with a 2- to 3-year interval was the most cost-effective strategy. As for the survival benefit between annual screening and interval screening, a prior meta-analysis showed that interval screening every 2-4 years was associated with a significantly lower risk of cancer-specific death (incidence rate ratio 0.79; 95% CI 0.69-0.91) than annual screening (incidence rate ratio 1.05; 95% CI 0.87-1.24) [12]. This study suggests a survival benefit of an extended-interval screening strategy, which is consistent with our findings.

In this study, we could not observe significantly lower risks of either PCa-specific mortality or all-cause mortality for people receiving serial screening at least once every 1-3 years. The main reason was cases with limited outcomes in these groups, resulting in relatively lower statistical power. To increase statistical power, participants were dichotomized on the basis of ever having undergone PSA screening once every 4 years. Expectedly, we observed a 70% decreased risk of PCa-specific death and a 23% decreased risk of all-cause death in this group. The result was robust against any unmeasured confounders (E-values are 6.12 for PCa death and 1.92 for overall death; Table S8 in Multimedia Appendix 1). This finding supports serial screening with at least 1 screening every 4 years to improve PCa survival among Chinese men.

Some limitations should be acknowledged in this study. First, selection, information, and immortal time biases were unavoidable in this cohort study, but a large sample size, rigorous analytic strategy, and protracted time frame of follow-up can increase the reliability of our findings. Second, information regarding covariates was poorly documented in the database, but we have used a multiple imputation approach to deal with the missing value. Third, unmeasured confounders may influence the accuracy of the estimates, but E-values were applied to assess the robustness of the results against unmeasured bias, making our findings more reliable and interpretable. Fourth, the limited number of cases of PCa onset and PCa death in the current data set led to insufficient statistical power to identify the optimal PSA screening interval. With longer-term follow-up available and more cases reaching the end point, a significant optimal screening interval would be identified in the future. Finally, the study population was derived from a single municipal district. The implementation of PSA screening and the incidence of PCa may vary greatly in different regions in China. Further studies based on a nationwide population with higher representativeness are warranted.

In conclusion, this large-scale population-based cohort study reveals a cumulative uptake rate of 17.9% for PSA testing, depicts an upward secular trend of screening attendance, and suggests an optimal screening frequency of at least 1 screening every 4 years for serial PSA testing in China. Findings of this study clarify the status of the PSA screening practice, strengthen the evidence base for an extended interval strategy for serial screening, and provide insights into the improvement of PCa survival for patients, health professionals, and policy makers.

## Appendix

Multimedia Appendix 1 Supplementary materials.

## Abbreviations

AAPC: average annual percent change
APC: annual percentage change
CHERRY: Chinese Electronic Health Records Research in Yinzhou
EHR: electronic health record
HR: hazard ratio
ICD-10: International Statistical Classification of Diseases, Tenth Revision
PA: physical activity
PCa: prostate cancer
PSA: prostate-specific antigen
PY: person-year
RCT: randomized controlled trial
WC: waist circumference

## Data Availability

Data of the Chinese Electronic Health Records Research in Yinzhou (CHERRY) study can be applied from the Health and Family Planning Bureau of Yinzhou District for health research purposes. More details are published on the study website [35].
