# Supplementary material for: The Impact of Prostate-Specific Antigen Screening on Prostate Cancer Incidence and Mortality in China: 13-Year Prospective Population-Based Cohort Study
Source: JMIR Public Health Surveill. 2024 Jan 18;10:e47161. doi: 10.2196/47161 (PMC10835592; doi:10.2196/47161)
Supplement: Multimedia Appendix 1 [file publichealth_v10i1e47161_app1.pdf]

Effects of prostate-specific antigen screening on prostate cancer incidence and mortality: a population-based cohort study in China

Supplementary materials:

Appendix 1. Data Quality Assessment Report 1

Table S1. Definitions of included variables 5

Table S2. Crude Cox regression model for PSA screening on PCa incidence 6

Table S3. Crude Cox regression model for PSA screening on PCa-specific mortality 7

Table S4. Crude Cox regression model for PSA screening on overall mortality 8

Table S5. Adjusted Cox regression model for PSA screening on PCa incidence 9

Table S6. Adjusted Cox regression model for PSA screening on PCa-specific mortality 11

Table S7. Adjusted Cox regression model for PSA screening on overall mortality . 13

Table S8. E-values for the primary effect sizes of PSA screening on PCa incidence and mortality 15

Figure S1. Directed acyclic graph (DAG) of PSA screening linking PCa incidence and mortality 16

Figure S2. Secular trends of the first PSA screening during 2010-2021 17

Appendix 1: Data Quality Assessment Report

We use a 3 × 3 matrix data quality assessment (DQA) framework proposed by Weiskopf[1], to assess the data quality of the present study. This framework allows researchers to easier decide whether the dataset, in particular, the electronic health record (EHR)-based dataset, is of sufficient quality through a logical and coherent check and report. Domain-specific assessments are summarized in Table 1 and detailed as follows.

Table 1. 3 × 3 DAQ framework for the present study

	A. Complete	B. Correct	C. Current
1. Patients	High quality	High quality	High quality
2. Variables	High quality	High quality	Medium quality
3. Time	High quality	High quality	Not applicable

High quality
Medium quality
Low quality
Not applicable

1A: There are sufficient data for each patient.

Assessment: High quality

Report: The core variables include uptake and date of prostate-specific antigen (PSA), first onset and date of prostate cancer (PCa), onset and date of death. 100% of subjects have data on ever PSA uptake. 5‰ of subjects have data on PCa diagnosis date while 95‰ of subjects are recorded for right censoring on PCa onset. 5% of subjects have data on mortality while 95% of subjects are recorded for right censoring on death (Figure 1).

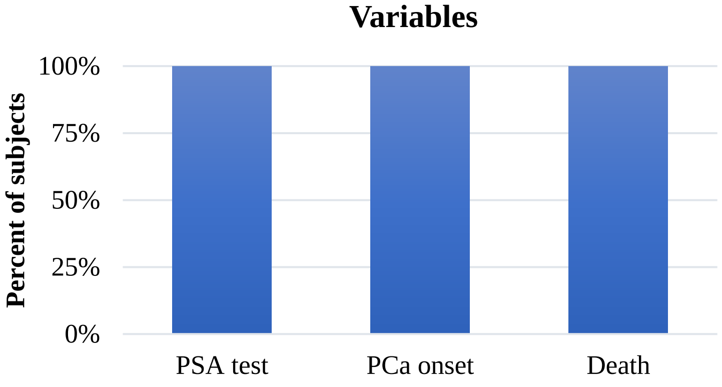

Figure 1. Percentage of subjects across core variables

2A: There are sufficient data for each variable.

Assessment: High quality

Report: The percentage of missing values for three core variables is 0%, while for other covariates are 0% for age, 0% for baseline PSA value, 3% for education level, 43% for marital status, 50% for body mass index, 48% for waist circumference, 46% for

smoking, 46% for drinking, 48% for physical activity, 35% for medication use, and 35% for comorbidity (Table 2). For missing values of the covariates, multiple imputation by chained equations (MICE) method is used to replace them. This method is commonly used for EHR-based studies, and has been proposed as standard imputation methods for missing values in the CHERRY study protocol.

Table 2. Missing proportions of variables of interests in this study

Variables	Missing (n, %)
Core variables	
PSA test	0 (0%)
PCa onset	0 (0%)
Death	0 (0%)
Covariates	
Age	0 (0%)
Baseline PSA value	0 (0%)
Education level	12,000 (3%)
Marital status	179,782 (43%)
Medication use	145,397 (35%)
Comorbidity	145,397 (35%)
Body mass index	210,431 (50%)
Waist circumference	200,706 (48%)
Smoking	191,625 (46%)
Drinking	191,978 (46%)
Physical activity	200,936 (48%)

3A: There are sufficient data for each time.

Assessment: High quality

Report: For this dimension, we do not require all variables except for uptake of PSA to be recorded in different time points. For patients with more than one time of PSA testing, all the records are completely recorded (100%).

1B: The distribution of values is plausible across patients.

Assessment: High quality

Report: As 45-year-old is the initiation age of PCa screening recommended by the National Cancer Center of China[2], we accordingly include male participants aged ≥45 years in the present study. The median age of the study subjects is 55 ys. The proportion of PSA uptake is 17.9%, which is consistent with prior Asian studies[3,4]. The distributions of other characteristic variables are plausible across patients.

2B: There is concordance between variables.

Assessment: High quality

Report: Two criteria are set to examine the concordance between variables in this study: (1) if PSA uptake=ever, then sex=male; (2) if number of PSA testing ≥1, then PSA uptake =ever. After checking, all the records containing PSA testing indicate that the subjects are male. All the records containing PSA testing with one time and above indicate that the subjects have ever PSA uptake. Thus, 100% of subjects have correct and concordance data according to the above criteria.

3B: The progression of data over time is plausible.

Assessment: High quality

Report: For this dimension, we do not require each variable to be recorded in different time points. For the overall dataset, we only expected one variable, the first uptake of PSA, to be cumulatively increased across year and Figure 2 shows the increment per expectation.

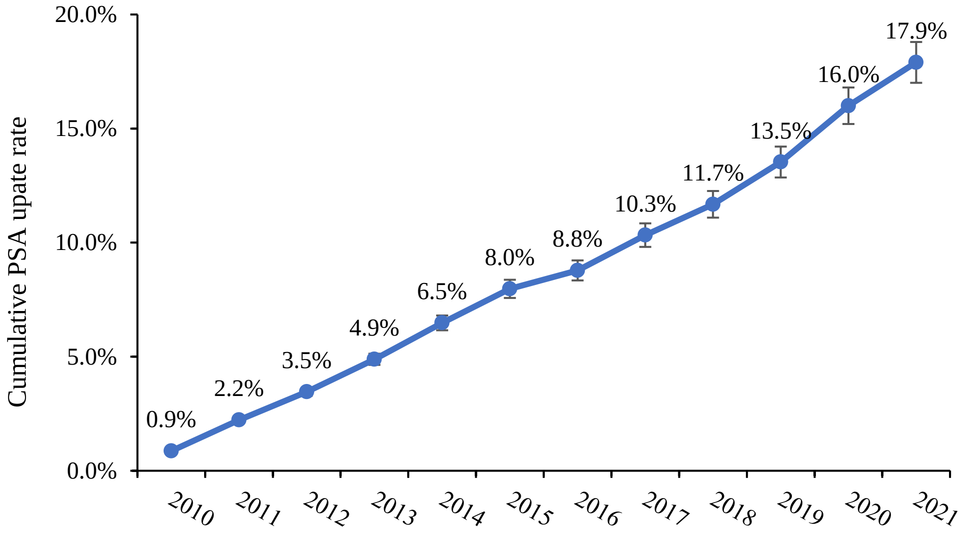

Figure 2. Cumulative PSA upate rate across year

1C: All data were recorded during the timeframe of interest.

Assessment: High quality

Report: The established timeframe for this study is between enrollment (Jan 1, 2009) and last follow-up (June 15, 2022). According to the DQA guideline, all demographic variables are not required to be recorded within the date range. Other data are restricted to the timeframe. 4% of subjects have recorded date of PSA test prior to the enrolment date. Considering the true exposure time of PSA test for this small proportion of subjects (<5%), we allow such deviation.

2C: Variables were recorded in the desired order.

Assessment: Medium quality

Report: Three criteria are set to examine the desired sequence between variables in this study: (1) enrolment date is prior to death date; (2) PCa diagnosis date is prior to death date; (3) No additional records should be generated follow by death. For criterion 1, 574 (0.1%) subjects whose recorded death date are prior to enrolment date (Jan 1, 2009) of this cohort. Such deviation is normal, because the electronic health system in Yinzhou is established before Jan 1, 2009, and subjects whose medical information are consecutively recorded since they consented to be recorded in the system. Thus, we exclude these ineligible subjects according to the inclusion criteria. For criterion 2, we do not detect any anomalies. For criterion 3, we detect 19 (<0.01‰) subjects with additional records (PSA test result date) after death date. After checking these 19 subjects, two reasons may explain such anomaly: (a) assuming a lag of 2-week of the lab value of PSA for those admitted endangered patients, PSA test result follows death by <2 weeks is plausible; (b) wrongly documented due to manual input. Data quality issues are nearly inevitable in up to 91.7% of HER-based and real-world study as reported[5]. Given a small proportion of such implausible documentation (<0.01‰) in our study, we believe the dataset is reliable and well-documented in general.

3C: Data were recorded with the desired regularity over time.

Assessment: N/A

Report: This dimension is not applicable for the present study, since we do not require any variables of interest to be recorded at regular intervals.

References:

[1] Weiskopf NG, Bakken S, Hripcsak G, et al. A Data Quality Assessment Guideline for Electronic Health Record Data Reuse. EGEMS (Wash DC). 2017 Sep 4;5(1):14.

[2] He J, Chen WQ, Li N, et al. China guideline for the screening and early detection of prostate cancer (2022, Beijing)]. Chin J Oncol. 2022,23;44(1):29-53.

[3] So WK, Choi KC, Tang WP, et al. Uptake of prostate cancer screening and associated factors among Chinese men aged 50 or more: a population-based survey. Cancer Biol Med 2014; 11(1): 56-63.

[4] Lin YH, Li HC, Chen HF, et al. Factors influencing southern Taiwanese men's acceptance of prostate - specific antigen screening. International Journal of Urological Nursing 2011; 5(2): 83-9.

[5] Edmondson ME, Reimer AP. Challenges frequently encountered in the secondary use of electronic medical record data for research. CIN: Computers, Informatics, Nursing. 2020 Jul 1;38(7):338-48.

Table S1. Definitions of included variables

Variables	Type	Definition	Response
**PSA screening**			
Ever screening	Binary	Ever have any PSA test or not	Never, ever
Number of PSA Screening	Continuous	A total count of screening during the follow-up years	For category use: 1, 2, ≥3
Screening frequency	Binary	Frequency of receiving one PSA test	At least annually/biennially/ triennially/quadrennially
Screening interval	Category	Average interval of having serial test during the follow-up	Irregular, 1-yr, 2-yr, 3-yr, 4-yr
Baseline PSA value, ng/mL	Continuous	PSA value at the first test	For binary use: <3, ≥3
Age at first PSA test, years	Continuous	Age at first PSA test	For binary use: <75, ≥75
**Outcomes**			
PCa incidence	Binary	Any incidence of PCa (ICD-C61)	No, yes
PCa-specific mortality	Binary	Any PCa death	No, yes
Overall mortality	Binary	Any death	No, yes
**Covariates**			
Socio-demographics			
Age, years	Continuous	Enrollment age	For category: 45-49, 50-54, 55-59, 60-64, 65-69, 70-74, ≥75
Education level	Binary	Highest degree obtained	Bachelors and above, below bachelors
Marital status	Categorical	Current marital status	Single, married, widowed, divorced
Anthropometry			
BMI (kg/m^2)	Continuous	Weight (kg) / square of height (m)	N/A
High WC	Binary	High if waist circumference ≥102 cm	No, yes
Lifestyle			
Tobacco smoking	Binary	Ever tobacco smoking	No, yes
Alcohol drinking	Binary	Ever alcohol drinking	No, yes
Physical activity	Categorical	Frequency of physical activity	Never, occasional (<1 per week), frequent (≥1 per week)
Medications			
Medication use	Binary	Any use of 5-alpha reductase inhibitors	No, yes
Comorbidities			
CCI	Counting	0, 1, 2, 3, 4, …	For binary use: 0, ≥1

PSA, prostate-specific antigen; PCa, prostate cancer, BMI, body mass index; WC, waist circumference; CCI, Charlson comorbidity index

Table S2. Crude Cox regression model for PSA screening on PCa incidence

	Cases	PYs	Incidence (/1,000 PYs)	HR (95%CI)
**PSA screening**				
Never	415	2,882,066	0.14	1.00
Ever	1,745	295,223	5.91	70.66 (57.18-87.33)
**Number of PSA screening***				
1	416	113,419	3.67	1.00
2	505	70,671	7.15	2.31 (1.98-2.68)
≥3	824	111,133	7.41	1.72 (1.48-2.00)
**At least annual screening***				
No	1,433	288,020	4.98	1.00
Yes	312	7,203	43.31	4.41 (3.83-5.09)
**At least biennial screening***				
No	1,129	238,450	4.73	1.00
Yes	616	56,774	10.85	1.46 (1.29-1.65)
**At least triennial screening***				
No	953	192,425	4.95	1.00
Yes	729	102,798	7.70	0.88 (0.78-1.00)
**At least quadrennial screening***				
No	877	162,773	5.39	1.00
Yes	868	132,450	6.55	0.61 (0.53-0.69)
**Screening interval***				
Irregular	877	162,773	5.39	1.00
1-yr	76	29,652	2.56	0.02 (0.01-0.04)
2-yr	176	46,024	3.82	0.09 (0.06-0.13)
3-yr	304	49,571	6.13	0.31 (0.25-0.39)
4-yr	312	7,203	43.32	2.76 (2.37-3.22)
**Baseline PSA value*, ng/ml**				
<3	309	251,221	1.23	1.00
≥3	1,436	44,002	32.63	69.18 (56.30-85.02)
**Age at first PSA test*, year**				
<75	1,288	248,200	5.19	1.00
≥75	457	47,023	9.72	2.20 (1.93-2.50)

PSA, prostate-specific antigen; PCa, prostate cancer; PY, person-years; HR, hazard ratio; CI, confidence interval

* Statistics were restricted to those ever-having PSA test

Table S3. Crude Cox regression model for PSA screening on PCa-specific mortality

	Cases	PYs	Mortality (/1,000 PYs)	HR (95%CI)
**PSA screening**				
Never	32	2,763,902	0.01	1.00
Ever	60	302,506	0.20	16.42 (10.45-25.78)
**Number of PSA screening***				
1	30	116,360	0.26	1.00
2	16	72,460	0.22	0.85 (0.47-1.57)
≥3	14	113,687	0.12	0.49 (0.26-0.92)
**At least annual screening***				
No	58	294,226	0.20	1.00
Yes	2	8,280	0.24	1.20 (0.29-4.97)
**At least biennial screening***				
No	50	243,817	0.21	1.00
Yes	10	58,689	0.17	0.76 (0.38-1.51)
**At least triennial screening***				
No	48	197,473	0.24	1.00
Yes	12	105,033	0.11	0.44 (0.23-0.83)
**At least quadrennial screening***				
No	48	167,703	0.29	1.00
Yes	12	134,803	0.09	0.30 (0.16-0.56)
**Screening interval***				
Irregular	48	167,703	0.29	1.00
1-yr	0	29,770	0.00	0.02 (0.01-0.04)
2-yr	2	46,344	0.04	0.09 (0.06-0.13)
3-yr	8	50,409	0.16	0.31 (0.25-0.39)
4-yr	2	8,280	0.24	2.76 (2.37-3.22)
**Baseline PSA value*, ng/ml**				
<3	9	252,300	0.04	1.00
≥3	51	50,206	1.02	28.33 (13.95-57.55)
**Age at first PSA test*, year**				
<75	20	253,329	0.08	1.00
≥75	40	49,177	0.81	10.27 (6.00-17.57)

PSA, prostate-specific antigen; PCa, prostate cancer; PY, person-years; HR, hazard ratio; CI, confidence interval

* Statistics were restricted to those ever-having PSA test

Table S4. Crude Cox regression model for PSA screening on overall mortality

	Cases	PYs	Mortality (/1,000 PYs)	HR (95%CI)
**PSA screening**				
Never	16,591	2,763,902	6.00	1.00
Ever	4,190	302,506	13.85	6.17 (5.79-6.57)
**Number of PSA screening***				
1	2,017	116,360	17.33	1.00
2	969	72,460	13.37	0.61 (0.55-0.69)
≥3	1,204	113,687	10.59	0.37 (0.33-0.42)
**At least annual screening***				
No	3,765	294,226	6.05	1.00
Yes	425	8,280	10.76	2.40 (2.11-2.74)
**At least biennial screening***				
No	3,027	243,817	12.42	1.00
Yes	1,163	58,689	19.82	0.75 (0.67-0.83)
**At least triennial screening***				
No	2,746	197,473	13.91	1.00
Yes	1,444	105,033	13.75	0.41 (0.37-0.45)
**At least quadrennial screening***				
No	2,646	167,703	15.78	1.00
Yes	1,544	134,803	11.45	0.29 (0.26-0.32)
**Screening interval***				
Irregular	2,646	167,703	15.78	1.00
1-yr	100	29,770	3.36	0.00 (0.00-0.01)
2-yr	281	46,344	6.06	0.03 (0.02-0.04)
3-yr	738	50,409	14.64	0.21 (0.18-0.24)
4-yr	425	8,280	51.33	1.41 (1.23-1.63)
**Baseline PSA value*, ng/ml**				
<3	3,137	252,300	12.43	1.00
≥3	1,053	50,206	20.97	1.68 (1.56-1.80)
**Age at first PSA test*, year**				
<75	1,807	253,329	7.13	1.00
≥75	2,383	49,177	48.46	6.65 (6.25-7.07)

PSA, prostate-specific antigen; PCa, prostate cancer; PY, person-years; HR, hazard ratio; CI, confidence interval

* Statistics were restricted to those ever-having PSA test

Table S5. Adjusted Cox regression model for PSA screening on PCa incidence

	Model I		Model II		Model III	
	HR (95%CI)	*P* value	HR (95%CI)	*P* value	HR (95%CI)	*P* value
**PSA screening**						
Never	1.00	Ref.	1.00	Ref.		
Ever	38.11 (33.86-42.91)	<.001	35.87 (31.82-40.85)	<.001		
**Number of PSA screening***						
1	1.00	Ref.	1.00	Ref.	1.00	Ref.
2	2.17 (1.91-2.48)	<.001	2.14 (1.88-2.46)	<.001	1.88 (1.65-2.14)	<.001
≥3	2.42 (2.16-2.72)	<.001	2.29 (2.05-2.59)	<.001	1.67 (1.48-1.88)	<.001
Continuous	1.05 (1.03-1.06)	<.001	1.04 (1.03-1.05)	<.001	1.01 (1.00-1.02)	0.13
**Screening frequency***						
At least annually	5.42 (4.74-6.21)	<.001	5.39 (4.71-6.17)	<.001	3.60 (3.15-4.10)	<.001
At least biennially	2.19 (1.97-2.42)	<.001	2.11 (1.90-2.33)	<.001	1.75 (1.57-1.93)	<.001
At least triennially	1.63 (1.49-1.79)	<.001	1.57 (1.43-1.73)	<.001	1.40 (1.28-1.55)	<.001
At least quadrennially	1.35 (1.23-1.49)	<.001	1.30 (1.19-1.43)	<.001	1.25 (1.13-1.36)	<.001
**Screening interval***						
Irregular	1.00	Ref.	1.00	Ref.	1.00	Ref.
1-yr	5.42 (4.68-6.29)	<.001	5.31 (4.57-6.11)	<.001	3.53 (3.03-4.06)	<.001
2-yr	1.26 (1.11-1.44)	<.001	1.20 (1.04-1.36)	.009	1.07 (0.94-1.22)	.29
3-yr	0.82 (0.71-0.96)	.01	0.79 (0.68-0.92)	.003	0.80 (0.69-0.94)	.006
4-yr	0.55 (0.44-0.69)	<.001	0.53 (0.43-0.67)	<.001	0.61 (0.49-0.76)	<.001

**Baseline PSA value*, ng/ml**				
<3	1.00	Ref.	1.00	Ref.
≥3	29.24 (25.70-33.28)	<.001	28.79 (25.28-32.79)	<.001
Continuous (log-transformed)	2.97 (2.87-3.08)	<.001	2.97 (2.86-3.06)	<.001
**Age at first PSA test*, year**				
<75	1.00	Ref.	1.00	Ref.
≥75	16.13 (11.92-21.84)	<.001	16.61 (12.18-22.42)	<.001
Continuous	1.48 (1.46-1.51)	<.001	1.49 (1.45-1.51)	<.001

PSA, prostate-specific antigen; PCa, prostate cancer; HR, hazard ratio; CI, confidence interval

* Statistics were restricted to those ever-having PSA test.

Model I: only stratification for age at PCa risk.

Model II: Model I + adjusted for education, marital status, BMI, High WC, smoking, drinking, physical activity, medication use, and comorbidity.

Model III: Model I + Model II + additionally adjusted for baseline PSA value and age at first PSA test.

Table S6. Adjusted Cox regression model for PSA screening on PCa-specific mortality

	Model I		Model II		Model III	
	HR (95%CI)	*P* value	HR (95%CI)	*P* value	HR (95%CI)	*P* value
**PSA screening**						
Never	1.00	Ref.	1.00	Ref.		
Ever	12.71 (8.31-19.45)	<.001	11.94 (7.69-18.54)	<.001		
**Number of PSA screening***						
1	1.00	Ref.	1.00	Ref.	1.00	Ref.
2	0.79 (0.43-1.45)	.46	0.79 (0.43-1.43)	.44	0.76 (0.42-1.40)	.04
≥3	0.43 (0.23-0.81)	.009	0.42 (0.22-0.79)	.007	0.36 (0.18-0.70)	.002
Continuous	0.77 (0.65-0.92)	.004	0.79 (0.65-0.95)	.01	0.74 (0.61-0.89)	.001
**Screening frequency***						
At least annually	1.32 (0.31-5.62)	.71	1.32 (0.31-5.58)	.71	0.76 (0.18-3.25)	.72
At least biennially	0.87 (0.43-1.77)	.70	0.86 (0.42-1.72)	.66	0.68 (0.33-1.38)	.29
At least triennially	0.47 (0.25-0.89)	.02	0.46 (0.24-0.87)	.02	0.41 (0.22-0.79)	.007
At least quadrennially	0.30 (0.16-0.58)	<.001	0.30 (0.16-0.57)	<.001	0.30 (0.16-0.57)	<.001
**Screening interval***						
Irregular	1.00	Ref.	1.00	Ref.	1.00	Ref.
1-yr	0.93 (0.22-3.97)	.92	0.91 (0.21-3.90)	.90	0.54 (0.13-2.32)	.41
2-yr	0.57 (0.27-1.23)	.16	0.56 (0.26-1.20)	.13	0.50 (0.23-1.08)	.08
3-yr	0.14 (0.03-0.59)	.007	0.14 (0.03-0.58)	.007	0.15 (0.04-0.62)	.009
4-yr	N/A	N/A	N/A	N/A	N/A	N/A

**Baseline PSA value*, ng/ml**				
<3	1.00	Ref.	1.00	Ref.
≥3	18.02 (8.44-38.47)	<.001	17.81 (8.41-38.09)	<.001
Continuous (log-transformed)	2.78 (2.49-3.11)	<.001	2.92 (2.64-3.22)	<.001
**Age at first PSA test*, year**				
<75	1.00	Ref.	1.00	Ref.
≥75	80.92 (17.70-369.97)	<.001	82.27 (18.17-376.15)	<.001
Continuous	1.31 (1.23-1.38)	<.001	1.13 (1.09-1.15)	<.001

PSA, prostate-specific antigen; PCa, prostate cancer; HR, hazard ratio; CI, confidence interval

* Statistics were restricted to those ever-having PSA test.

Model I: only stratification for age at PCa death risk.

Model II: Model I + adjusted for education, marital status, BMI, High WC, smoking, drinking, physical activity, medication use, and comorbidity.

Model III: Model I + Model II + additionally adjusted for baseline PSA value and age at first PSA test.

Table S7. Adjusted Cox regression model for PSA screening on overall mortality

	Model I		Model II		Model III	
	HR (95%CI)	*P* value	HR (95%CI)	*P* value	HR (95%CI)	*P* value
**PSA screening**						
Never	1.00	Ref.	1.00	Ref.		
Ever	1.70 (1.64-1.76)	<.001	1.27 (1.20-1.35)	<.001		
**Number of PSA screening***						
1	1.00	Ref.	1.00	Ref.	1.00	Ref.
2	0.77 (0.72-0.83)	<.001	0.78 (0.72-0.84)	<.001	0.82 (0.76-0.89)	<.001
≥3	0.63 (0.59-0.67)	<.001	0.64 (0.60-0.69)	<.001	0.72 (0.67-0.77)	<.001
Continuous	0.95 (0.93-0.96)	<.001	0.95 (0.93-0.97)	<.001	0.97 (0.95-0.99)	.001
**Screening frequency***						
At least annually	3.20 (2.88-3.55)	<.001	3.25 (2.92-3.60)	<.001	2.68 (2.41-2.97)	<.001
At least biennially	1.54 (1.44-1.65)	<.001	1.55 (1.45-1.67)	<.001	1.39 (1.30-1.49)	<.001
At least triennially	0.99 (0.93-1.05)	.66	1.00 (0.94-1.07)	.93	0.97 (0.90-1.03)	.28
At least quadrennially	0.74 (0.70-0.79)	<.001	0.76 (0.71-0.81)	<.001	0.77 (0.73-0.82)	<.001
**Screening interval***						
Irregular	1.00	Ref.	1.00	Ref.	1.00	Ref.
1-yr	2.72 (2.44-3.03)	<.001	2.80 (2.51-3.13)	<.001	2.36 (2.11-2.64)	<.001
2-yr	0.95 (0.88-1.03)	.23	0.97 (0.90-1.05)	.45	0.91 (0.84-0.99)	.03
3-yr	0.40 (0.35-0.45)	<.001	0.41 (0.36-0.46)	<.001	0.43 (0.38-0.48)	<.001
4-yr	0.22 (0.18-0.27)	<.001	0.23 (0.19-0.28)	<.001	0.26 (0.21-0.32)	<.001

**Baseline PSA value*, ng/ml**				
<3	1.00	Ref.	1.00	Ref.
≥3	1.05 (0.98-1.13)	.18	1.07 (0.99-1.15)	.08
Continuous (log-transformed)	0.98 (0.96-1.01)	.25	0.99 (0.96-1.02)	.42
**Age at first PSA test*, year**				
<75	1.00	Ref.	1.00	Ref.
≥75	20.97 (17.70-24.83)	<.001	19.89 (16.78-23.81)	<.001
Continuous	1.37 (1.35-1.40)	<.001	1.39 (1.36-1.42)	<.001

PSA, prostate-specific antigen; PCa, prostate cancer; HR, hazard ratio; CI, confidence interval

* Statistics were restricted to those ever-having PSA test.

Model I: only stratification for age at death risk.

Model II: Model I + adjusted for education, marital status, BMI, High WC, smoking, drinking, physical activity, medication use, and comorbidity.

Model III: Model I + Model II + additionally adjusted for baseline PSA value and age at first PSA test.

Table S8. E-values for the primary effect sizes of PSA screening on PCa incidence and mortality

	PCa incidence		PCa-specific mortality		Overall mortality	
	E-value	LL / UL	E-value	LL / UL	E-value	LL / UL
**Number of PSA screening**						
1	1.00	1.00	1.00	1.00	1.00	1.00
2	3.17	2.69	1.96	1.00	1.74	1.50
≥3	2.73	2.32	5.00	2.21	2.12	1.92
Continuous	1.11	1.00	2.04	1.50	1.21	1.11
**Screening frequency**						
At least annually	6.66	5.75	1.96	1.00	4.80	4.25
At least biennially	2.90	2.52	2.30	1.00	2.13	1.92
At least triennially	2.15	1.88	4.31	1.85	1.21	1.00
At least quadrennially	1.81	1.51	6.12	2.90	1.92	1.74
**Screening interval**						
Irregular	1.00	1.00	1.00	1.00	1.00	1.00
1-yr	6.52	5.51	3.11	1.00	4.15	3.64
2-yr	1.34	1.00	3.41	1.00	1.43	1.11
3-yr	1.81	1.32	12.81	2.61	4.08	3.59
4-yr	2.66	1.96	N/A	N/A	7.15	5.70
**Frequent screening**						
No	1.00	1.00	1.00	1.00	1.00	1.00
Yes	2.15	1.88	4.31	1.85	1.21	1.00

PSA, prostate-specific antigen; PCa, prostate cancer; LL, E-value for the lower limit of the confidence interval; UL, E-value for the upper limit of the confidence interval

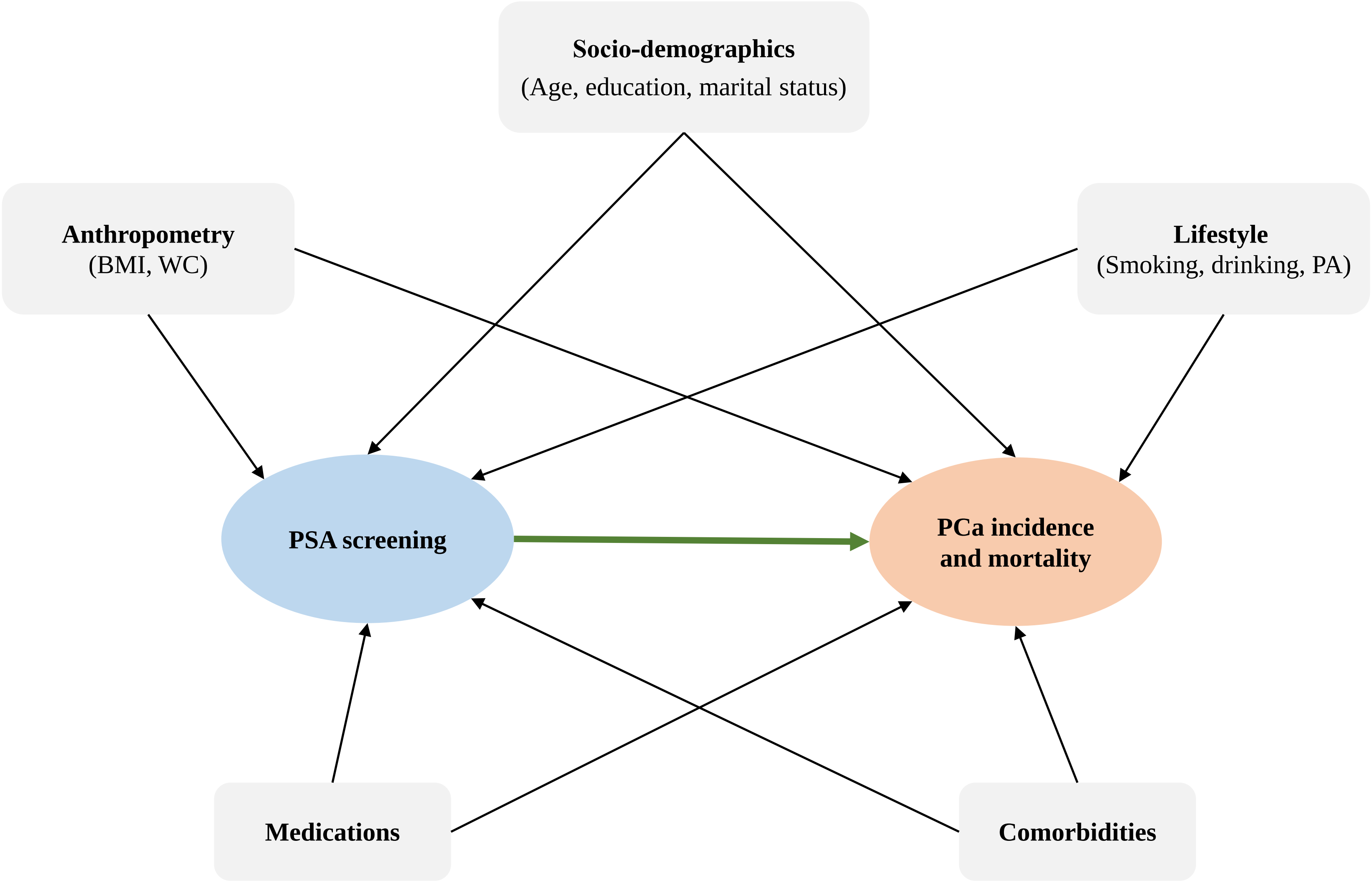

Figure S1. Directed acyclic graph (DAG) of PSA screening linking PCa incidence and mortality

Exposure Outcome Confounder Casual path Bias path

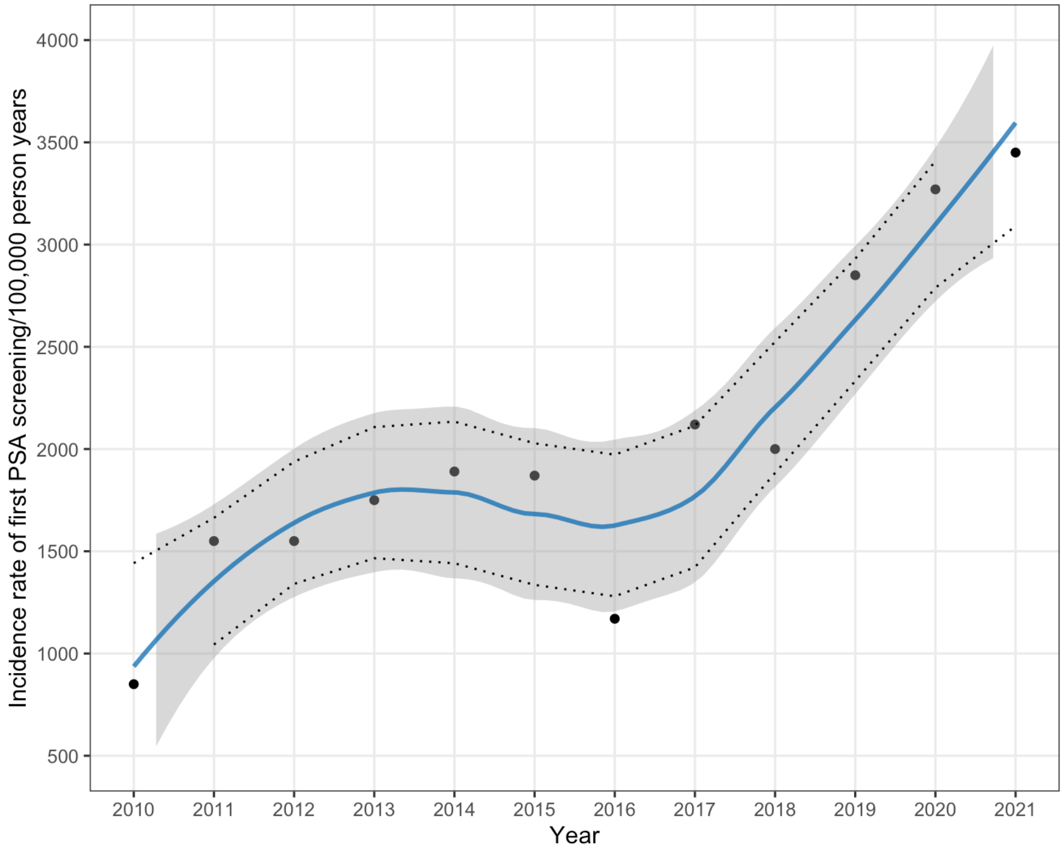

Timeframe	APC, % (95%CI)	*P* value
2010-2016	3.23 (-5.58 to 12.86)	0.427
2016-2021	15.65 (7.97 to 23.88)	0.002
2010-2021	8.70 (3.61 to 14.04)	0.001

Figure S2. Secular trends of the first PSA screening during 2010-2021
